# Implementing STEADI in Academic Primary Care to Address Older Adult Fall Risk

**DOI:** 10.1093/geroni/igx028

**Published:** 2017-11-28

**Authors:** Elizabeth Eckstrom, Erin M Parker, Gwendolyn H Lambert, Gray Winkler, David Dowler, Colleen M Casey

**Affiliations:** 1 Department of Medicine, Division of General Internal Medicine and Geriatrics, Oregon Health & Science University, Portland; 2 U.S. Public Health Service, National Center for Injury Prevention and Control, Centers for Disease Control and Prevention, Atlanta, Georgia; 3 Program Design and Evaluation Services, Multnomah County Health Department and Oregon Public Health Division, Portland; 4 Providence Health & Services, Portland, Oregon

**Keywords:** Clinical practice, Falls, Information technology, Intervention

## Abstract

**Background and Objectives:**

Falls are the leading cause of injury-related deaths in older adults. Objectives include describing implementation of the Centers for Disease Control and Prevention’s Stopping Elderly Accidents, Deaths, and Injuries (STEADI) initiative to help primary care providers (PCPs) identify and manage fall risk, and comparing a 12-item and a 3-item fall screening questionnaire.

**Design and Methods:**

We systematically incorporated STEADI into routine patient care via team training, electronic health record tools, and tailored clinic workflow. A retrospective chart review of patients aged 65 and older who received STEADI measured fall screening rates, provider compliance with STEADI (high-risk patients), results from the 12-item questionnaire (*Stay Independent*), and comparison with a 3-item subset of this questionnaire (*three key questions*).

**Results:**

Eighteen of 24 providers (75%) participated, screening 773 (64%) patients over 6 months; 170 (22%) were high-risk. Of these, 109 (64%) received STEADI interventions (gait, vision, and feet assessment, orthostatic blood pressure measurement, vitamin D, and medication review). Providers intervened on 85% with gait impairment, 97% with orthostatic hypotension, 82% with vision impairment, 90% taking inadequate vitamin D, 75% with foot issues, and 22% on high-risk medications. Using *three key questions* compared to the full *Stay Independent* questionnaire decreased screening burden, but increased the number of high-risk patients.

**Discussion and Implications:**

We successfully implemented STEADI, screening two-thirds of eligible patients. Most high-risk patients received recommended assessments and interventions, except medication reduction. Falls remain a substantial public health challenge. Systematic implementation of STEADI could help clinical teams reduce older patient fall risks.

**Translational Significance**

Falls are the leading cause of injury-related deaths in older adults. After embedding the Centers for Disease Control and Prevention’s Stopping Elderly Accidents, Deaths, and Injuries (STEADI) protocol into the clinic workflow and electronic health record, primary care providers implemented preventive interventions for patients at high risk for future falls. Interventions were directed toward more than 80% of patients with gait or vision impairment, orthostasis, or vitamin D deficiency. Comparison of a 3-item and 12-item screening questionnaire showed that the briefer version could be effective and more efficient for screening for falls. Systematic implementation of STEADI could help clinical teams reduce older patient fall risks.

Falls are the leading cause of injury-related deaths in older adults, accounting for nearly 3 million emergency department visits, including 925,000 hospitalizations, and more than 28,000 deaths in 2015 in the United States (WISQARS, 2016). Fallers often experience decreased mobility, independence, and fear of falling, which predispose them to future falls. Worse, death rates from falls doubled between 2000 and 2014, from 29 to 58/100,000 population (WISQARS, 2016). Falls result in over $31 billion in medical costs each year (Burns, Stevens, & Lee, 2016). Although not all risk factors for falls are modifiable (age, some chronic illnesses and physical limitations), a systematic review of fall prevention interventions for community-dwelling older adults found falls may be decreased by programs that target gait, strength, and balance (e.g., Tai Chi), home safety, gradual withdrawal of high-risk medications, and other interventions (Gillespie et al., 2012).

To address this growing public health epidemic, the Centers for Disease Control and Prevention (CDC) developed the Stopping Elderly Accidents, Deaths, and Injuries (STEADI) initiative to facilitate fall risk identification and management in primary care (Stevens & Phelan, 2013). Development of STEADI was informed by the American and British Geriatric Societies’ (AGS/BGS) 2010 fall prevention guideline (Kenny, Rubenstein, Tinetti, Brewer & Cameron, 2011) as well as two conceptual models—Wagner’s Chronic Care model (Wagner, 1998) and Prochaska’s Transtheoretical Stages of Change model (Prochaska & Velicer, 1997). Wagner’s Chronic Care model focuses on changes that are needed for clinical systems that have been developed to deal with acute problems to “reconfigure themselves specifically to address the needs and concerns of chronically ill patients,” which require “planned regular interactions with their caregivers, with a focus on function and prevention of exacerbations and complications” (Wagner, 1998). In STEADI, fall risk is conceptualized as a chronic illness, as steps to address underlying health issues and prevent falls require a similar reorganization of health care system processes and regular patient/provider interactions over an extended time period. Following Prochaska’s Stages of Change model, STEADI is built on the idea that (1) fall prevention requires health behavior change, (2) behavior change is a process that occurs through a series of stages, and (3) fall prevention interventions should be tailored to a patient’s stage of change (Prochaska & Velicer, 1997). Thus, STEADI posits that a provider’s interactions with a patient should be guided by the stage at which a patient presents—precontemplation, contemplation, preparation, or action (Stevens & Phelan, 2013). STEADI was further refined by focus groups with health care providers, which informed application of these models into practice (Stevens & Phelan, 2013). A comprehensive description of the development of STEADI is available elsewhere (Stevens & Phelan, 2013).

STEADI includes a suite of materials to help primary care teams implement the 2010 AGS/BGS fall prevention clinical practice guidelines (Kenny et al., 2011). Available at www.cdc.gov/steadi, STEADI includes: (1) a 12-question patient screening questionnaire of fall risk factors (*Stay Independent*); (2) an algorithm to guide clinical teams on how to assess and manage fall risk (see Supplementary Figure 1); (3) educational materials for providers, including case studies, conversation starters, online trainings, and standardized gait and balance assessments with instructional videos; and (4) educational brochures for older adults and their caregivers. The goal of STEADI is to increase the skills of primary care providers (PCPs) and their teams to systematically screen older patients for fall risk, assess whether patients have modifiable fall risk factors, and treat the identified risk factors using evidence-based interventions.

The first step in a multifactorial clinical fall prevention approach is fall risk screening to identify older adults who are at increased risk of falling. The initial screening step is critical because it identifies who will receive additional assessments and follow-up care. There is currently no standard for outpatient fall risk screening; those implementing clinical fall prevention typically use a variety of tools to identify who may be at risk (Close & Lord, 2011; Gates, Smith, Fisher, & Lamb, 2008). The STEADI initiative includes information on two screening options. A 12-item patient questionnaire, called the *Stay Independent,* has been validated to a clinical examination (Rubinstein et al., 2011). The *Stay Independent* can be used as a screening questionnaire, with a score of four or more indicating increased risk of falling; furthermore, responses to individual questions can point to specific risk factors and clinical issues that may require additional follow-up (Rubinstein et al., 2011).

To reduce the amount of time it takes to screen patients, the STEADI initiative also describes how three key questions could be used to screen for fall risk. Clinicians ask their patients “have you fallen in the last year, do you feel unsteady when standing or walking, and do you worry about falling?” These questions, a subset of concepts included in the full Stay Independent, focus on two of the biggest risk factors for falling (history of falls and gait/strength/balance), and align with the screening questions recommended by the AGS/BGS guideline (Kenny et al., 2011). Worry about falling was also included because fear of falling has been linked to falling (Delbaere, Crombez, Vanderstraeten, Willems, Cambier, 2004) and has been shown to be related to gait issues even in the absence of a history of falls (Makino et al., 2017). Worrying about falling may indicate that the older adult is in the “preparation stage” of the Stages of Change model (Prochaska & Velicer, 1997), and thus may be amenable to making changes to address their fall risk. In order to ensure that at-risk older adults are not missed, providers using the three key question approach are asked to follow up with patients that responded yes to *any* of the three key questions. The implementation of STEADI at OHSU, which implemented the full *Stay Independent* brochure, provides an opportunity to assess some implications of using the three key questions rather than the complete Stay Independent brochure. This information is useful to providers when determining which approach to use. We hypothesized that use of three key questions would find at least as many older adults at risk for falls as the use of the full questionnaire would identify.

Electronic health records (EHRs) are widely used in health care settings, and there is emerging evidence that EHRs can facilitate assessment and management of chronic health conditions (Loo et al., 2011; Schnipper et al., 2010; Spears et al., 2013). Building fall prevention tools into EHR systems and clinic workflows could help make fall prevention a routine part of clinical practice. To this end, the Internal Medicine and Geriatrics Clinic at Oregon Health & Science University (OHSU) modified their Epic EHR tools and clinic workflow to integrate STEADI. Lessons learned at OHSU during STEADI implementation are described elsewhere (Casey et al., 2016). Objectives for this study were to report on STEADI implementation, including the care received by patients identified as high-risk for falling, and to compare the full 12-item *Stay Independent* with a briefer *three key question* subset of this questionnaire, to evaluate whether a shorter questionnaire could adequately identify high-risk patients.

## Research Design and Methods

This study to evaluate the implementation of a new evidence-based practice protocol occurred in two phases. During the initial implementation phase (March 31 to June 8, 2014), the STEADI protocol and EHR tools were tested and updated multiple times to improve and streamline the process, including changing data entry of the *Stay Independent* score from a binary “low” versus “high” risk to recording all 12 item-level responses. Full implementation occurred after these improvements were adopted (June 9, 2014 and after). The OHSU Institutional Review Board approved the project.

### Subjects

Patients aged 65 and older were eligible for STEADI unless they had a diagnosis of dementia or “frequent falls” (since this was a screening study), were receiving hospice care, or were nonambulatory. Eligible patients had an office visit with a PCP who was participating in the project during the study time period, and had not previously had a fall screening in the prior calendar year.

### Team Training

A voluntary group of OHSU internal medicine and geriatric PCPs were recruited to participate in the project and took part in a 1-hour training session, which provided information on how to use the STEADI workflow and EHR tools. They were incentivized to participate in the study by being able to receive credit for participation toward Maintenance of Certification through the American Board of Internal Medicine. STEADI intervention leaders—called “STEADI champions” (EE and CMC)—delivered separate trainings to providers and staff to educate them on the STEADI protocol, EHR tools, and workflow. Training for providers focused on how to apply the EHR tools to help guide interventions during the office visit. Staff training focused on the clinic workflow, including how to correctly take orthostatics and perform the Timed Up and Go test. The champions also conducted weekly feedback sessions and two “brown bag” lunch refresher trainings to target areas of concern from PCPs and staff.

### EHR Tools and Clinic Workflow

Informatics staff built STEADI elements into an EHR (Epic) clinical decision support tool to help the clinical workflow align with the STEADI algorithm (see Supplementary Figure 1). To simplify integration, STEADI tools mirrored EHR technology already being used, including developing an annual fall “health maintenance modifier” and a STEADI “Smartset” containing standardized note templates (“dotphrases”), data entry tables (“docflowsheets”), checklists for orders and diagnostic codes, and Current Procedural Terminology II (CPT II) codes to report on fall-related national quality measures (Casey et al., 2016). Content from CDC-developed patient educational brochures was embedded into the STEADI “Smartset” to include in patients’ after visit summaries. STEADI champions worked closely with an informatics staff assigned to this project to create, test, and review iterative versions of the STEADI EHR tool before full implementation. All EHR tools have now been published as an Epic Clinical Program, which includes an instruction manual for EHR analysts to build the tools into their own system.

Every eligible patient had a fall “health maintenance modifier” added to their chart at the beginning of the study. Eligible patients’ lists of health maintenance modifiers included “Fall Screening Due.” These modifiers were routinely reviewed by the medical assistants before each day’s appointments to identify any necessary health screenings due (e.g., falls, mammography). If a fall screening was due, the medical assistant would add “Fall Screening” to the patient’s appointment notes so it would be seen by the front office staff. If an eligible patient came in for an office visit or Medicare Wellness Visit with their PCP and their appointment notes indicated they were due for a fall screening, the front office staff gave the patient the 12-question *Stay Independent* questionnaire at check-in to start the clinic workflow. The patient independently completed the paper questionnaire in the waiting room. When the medical assistant roomed the patient, they reviewed the questionnaire and tallied the positive responses, and entered this score into the EHR’s STEADI “docflowsheet.” A *Stay Independent* score of four or higher indicated high-risk for falls and a score of three or less indicated low-risk (Rubenstein et al., 2011). Although the STEADI algorithm delineates a moderate risk category based on number of falls or injury related to a fall, for purposes of clinical feasibility, our study used only low- and high-risk categories based solely on the score of the STEADI questionnaire.

If low-risk, the medical assistant entered the score and gave the patient a handout on home safety and other fall prevention strategies at the beginning of the visit. If high-risk, the medical assistant completed a Timed Up and Go walking test and Snellen vision test on the way to the exam room. Once in the exam room, the medical assistant performed orthostatic vital signs as part of the rooming process and entered all data into the EHR (Kalinowski, 2008; Podsiadlo & Richardson, 1991). This front-end risk stratification into high- and low-risk allowed PCPs to have the timed walking test, vision, and orthostatic data early in their visit, eliminating the need for additional testing later.

For patients receiving a full STEADI evaluation because their STEADI score was 4 or more, the PCP would open the STEADI “Smartset” within the EHR as part of the visit. This “Smartset” provided access to pertinent orders, the note template, and all fall-related patient education materials within a single location. The PCP reviewed the results of the Timed Up and Go, vision assessment, and orthostatics. If impairment was present, the PCP recommended interventions such as physical therapy referral or Tai Chi, referral to an ophthalmologist, or adjustment of blood pressure medications and improved hydration, respectively. The PCP also determined whether the patient was on adequate vitamin D based on past laboratory levels (if available) and medication list or patient report of daily vitamin D dose. A footwear assessment included a monofilament exam or review of last monofilament exam if the patient was diabetic; for nondiabetic patients, the PCP evaluated whether the patient generally wore appropriate footwear (e.g., no flip flops, no bare feet at home, no high heels) and made appropriate recommendations. The medication list was initially reviewed by the medical assistant, but the PCP was trained to pay special attention to any high-risk medications (National Guideline Clearinghouse, 2015) and to intervene for a high-risk medication by eliminating, tapering the dose, or substituting the medication with a safer alternative (clinic workflow previously published, see Casey, et al., 2017).

When PCPs felt their schedules were too busy, they could request the MA remove the STEADI “flag” and patients would not be given the *Stay Independent* questionnaire at check-in, thus deferring the screening until a later date. If a patient screened high-risk, but the PCP did not have time to complete additional STEADI fall risk assessments and interventions, usually because of competing medical priorities, the PCP could “defer” the full evaluation until a later date. PCPs would instruct front desk staff in a patient’s check out note to reschedule the patient for a STEADI follow up appointment and include “STEADI follow up” in the appointment notes. That patient would not need to complete the STEADI questionnaire again at the future appointment.

## Data Collection and Analysis

### Study Sample and Data Collection

Chart review was conducted on a subset (405) of the 773 eligible patients who received STEADI from June 9 through December 31, 2014. We reviewed all charts of patients identified as high risk based on either the *Stay Independent* (170 patients) or *three key questions* (an additional 111 patients) and used a 1:4 sampling ratio for chart reviews of patients who were low-risk based on both questionnaires (reviewed 124 patient charts of 492 who screened low-risk).

#### Variables

Abstracted data included gender, PCP name, age, race/ethnicity, comorbidities, the *Stay Independent* questionnaire total score and item-level responses to each of the 12 questions. All variables were recorded based on previous documentation in the chart; no new variables were collected from the patient outside of the STEADI questionnaire and other visit-related parameters. Comorbidities were coded as present or absent and were based on whether the disease was listed on the problem list, including arthritis, vision problems, stroke, congestive heart failure, chronic obstructive pulmonary disease, chronic pain, depression, diabetes, incontinence, muscle weakness, gait abnormality, use of assistive device, and cognitive impairment. Cognitive impairment included both mild cognitive impairment as well as any dementia diagnosis. Hypotension or orthostatic hypotension were defined based on chart review for the prior year during which time a patient had at least one measurement of blood pressure less than 120 mm Hg systolic or a difference in systolic blood pressure of 20 points when orthostatic blood pressure was measured. All present comorbidities were then summed for each patient to establish a comorbidity “profile.”

Data abstraction also included all interventions provided to patients who scored high-risk (score ≥ 4) on the *Stay Independent* questionnaire as previously described in the description of the study’s workflow (e.g., administration of the Timed Up and Go test, orthostatic blood pressure measurements, vision screening, evaluation of feet problems, medication review). Each assessment variable was recorded as completed or not completed by the appropriate team member (e.g., medical assistant for orthostatic vital signs, PCP for vitamin D status); and if assessed, binary data entered as to whether there was impairment or not. Furthermore, if impairment was identified, binary data recorded whether an intervention was recommended for each issue identified. For medication review and medication-related interventions, interventions were coded as ‘medication changed;’ ‘no changes made, patient preference;’ ‘medication change deferred; rationale provided.’ This coding scheme applied to each medication if the patient took multiple high-risk medications.

#### Comparison of 12-item questionnaire versus 3-item subset

We compared fall risk based on the total 12-item *Stay Independent* questionnaire score to an affirmative response to any one of *three key questions* (a subset of *Stay Independent*): Have you fallen in the past year? Do you feel unsteady when standing or walking? Do you worry about falling? This briefer version of the *Stay Independent* questionnaire could reduce the burden of screening for patients and clinic teams. All screened patients were allocated into four categories based on their responses to the *Stay Independent* questionnaire: two concordant groups (high-risk using both approaches and low-risk using both approaches) and two discordant groups (high-risk using one approach and low-risk using the other). We described the distribution across the four groups for the entire sample, and compared the characteristics across these four groups. Results for the total group were weighted to account for the one in four sampling of patients in the concordant low category.

#### Statistics

Data were entered into an Excel spreadsheet and then transferred to IBM SPSS statistics software (version 23) for analysis. We used descriptive statistics to compare the characteristics of screened patients in the two separately identified high-risk groups (those that scored high risk on the *Stay Independent* regardless of score on the *three key questions* and those that scored high risk on the *three key questions* but not the full *Stay Independent*) to the concordant low-risk group (those that scored low risk using both approaches). *T*-tests were used for testing mean differences (for continuous variables) and chi-square was used to test differences between proportions.

## Results

### Fall Screening Rates

Eighteen providers (of 24, 75%) participated in STEADI and saw 1,495 patients aged 65 and older. No demographic information was collected on providers who chose not to participate in STEADI. We excluded 288 patients (19%) due to a prior diagnosis of frequent falls, dementia, being nonambulatory, or on hospice. Of the remaining 1,207 eligible patients, 773 (64%) completed the *Stay Independent* questionnaire. Thirty-six percent of eligible patients were not screened with the Stay Independent questionnaire because their provider had felt there was not time at that visit to do the screening. Seventy-three percent of STEADI visits occurred as part of routine office visits, 25% occurred during Medicare Wellness Visits, and 2% occurred during new patient visits. Of the 773 screened patients, 603 (78%) patients screened at low-risk for falls, and 170 (22%) screened at high-risk for falls based on the *Stay Independent* questionnaire (Table 1).

**Table 1.** Fall Screening Questionnaire Results for Patients Aged 65 and Older, and Comparison of 12-Item “Stay Independent” Questionnaire and Three Key Questions (2014) Columns Are the Results of Full STEADI Screening

Answers to three key questions	Low-risk total score (score < 4)	High-risk total score (score ≥ 4)	Total patients by risk
Low-risk (no to all three questions)	Concordant low-risk^a^, *A* = 492^b^	Discordant (stay independent = high-risk)^c^, *B* = 9	*A* + *B* = 501 (98% concordance)
High-risk (yes to at least one question)	Discordant (key questions = high- risk)^d^, *C* = 111	Concordant high-risk^e^, *D* = 161	*C* + *D* = 272 (59% concordance)
Total patients by score	A + C = 603	B + D = 170	A + B + C + D = 773 (84% concordance overall)

*Note:* The *Three Key Questions* of the *Stay Independent* Questionnaire are; 1. Have you fallen in the past year?; 2. Do you feel unsteady when standing or walking?; 3. Do you worry about falling?

^**a**^Both screening approaches indicate patient is low-risk.

^b^Chart review was done on sample of 124 of these 492 low-risk patients.

^c^ *Stay Independent* indicates patient at high-risk; *three key questions* indicate low-risk.

^d^ *Three key questions* indicate patient at high-risk; *Stay Independent* indicates low-risk.

^e^Both screening approaches indicate patient is at high-risk.

### Fall Prevention Interventions Received by Patients at High-Risk for Falls

**Figure 1.**
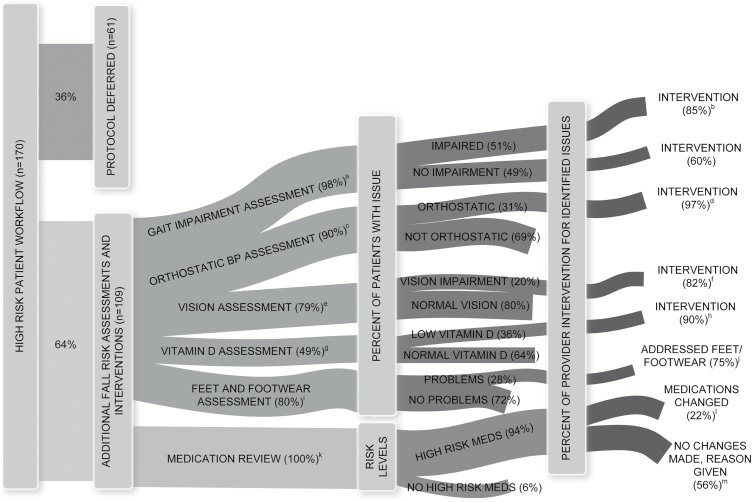
Percent of patients at a high risk for falls by the “Stay Independent” questionnaire who received each intervention. ^a^Gait impairment assessment consisted of Timed-Up-and-Go testing, with a score greater than 15 seconds or current use of mobility aid indicating impairment. ^b^Gait impairment interventions included: home safety evaluation, exercise recommendation, mobility aid evaluation, physical or occupational therapy, Tai Chi, falls prevention class, Otago referral, pelvic floor therapy, or patient declined intervention. ^c^Orthostatic blood pressure (BP) assessment consisted of two consecutive BP measurements, lying for 5 minutes and then standing for one minute, with orthostatic BP defined as a drop of 20 points or greater in systolic BP. ^d^Orthostatic blood pressure interventions included: goal BP discussed, medication management, hydration addressed, compression stockings advised, education provided on position changes, self-monitoring of home BP. ^e^Vision assessment consisted of Snellen vision testing, with acuity worse than 20/40 indicating poor vision. ^f^Vision interventions included: consult to ophthalmology or optometry, already seeing ophthalmologist or optometrist, recommendation for single distance lenses outdoors. ^g^Vitamin D assessment consisted of lab testing of vitamin D serum 25(OH) levels within last 12 months, with values <30 nmol/L (<12 ng/mL) considered low. ^h^Vitamin D interventions included: review of patient’s current supplements and increase in dosage or new prescription for vitamin D if needed. ^i^Feet or footwear assessment consisted of clinical evaluation of feet and footwear, review of monofilament testing of diabetic patient. ^j^Feet or footwear interventions included: consult to podiatry, counseled and footwear handout provided, physical therapy. ^k^High-risk medication review consisted of reviewing medication list during visit for the following: benzodiazepines, other anxiolytic, selective serotonin reuptake inhibitors/serotonin and norepinephrine reuptake inhibitors, tricyclic antidepressants, monoamine oxidase inhibitors, antipsychotic medication, alternative antidepressants, seizure medication, lithium, diuretics, beta blockers, angiotensin-converting enzyme inhibitors/angiotensin II receptor blockers, calcium channel blockers, systemic glucocorticoids, anticholinergics, antihistamines, carbidopa/levodopa, opioids. ^l^High-risk medication changes included: titration, dose reduction or discontinuation of high-risk medication, no changes made (reason given). ^m^Reasons for no changes made: patient preference not to change medication, risk versus benefit discussion, referral for Nurse Care Manager (NCM) visit for medication review, hold for more data (labs, BP), have titrated medications in the past without benefit.

Of the 170 patients screened as high-risk using the 12 *Stay Independent* questionnaire, 109 (64%) received additional fall risk assessments and interventions, whereas the remaining 36% had their fall prevention intervention deferred (Figure 1). Providers completed appropriate interventions for 85% of patients with gait impairment, 97% with orthostasis, 82% with vision impairment, 90% with vitamin D deficiency, and 75% with foot or footwear issues. Of the 94% of patients who were on one or more high-risk medications, at least one medication was tapered for 22% of patients, and rationale was provided for not tapering high-risk medications in 56%. Providers referred 60% of high-risk patients *without* gait impairment for community tai chi or fall prevention classes to help prevent future gait and balance issues (data not shown). For 61 (36%) high-risk patients, the provider “deferred” further assessment to a future office visit, usually due to lack of time. Most “deferred” patients did not have further fall assessment during the study period.

### Comparison of Questionnaire Versions

The 12-item *Stay Independent* questionnaire classified 170 (22%) patients as high-risk based on a score of 4 or more. Of these patients, 161 (95%) would have been identified as high-risk using an affirmative response to any one of the *three key questions*. An additional 111 patients would have been high-risk using the *three key questions* (Table 1). Only nine patients who screened high-risk using the *Stay Independent* questionnaire were categorized as low-risk using only the *three key questions* (these nine patients were analyzed in the high-risk group for purposes of data analysis).

Several significant differences (*p* < .05) emerged for patients who scored low-risk using both approaches compared to those who scored high-risk using either approach (Table 2). Low-risk patients were, on average, younger (mean age 71.8 vs 73.5 based on 3-item only vs 76.5 based on 12-item). Low-risk patients had fewer comorbid conditions (1.8 vs 2.3 vs 3.8 for the respective approaches; maximum reported comorbidities for any individual was 7). Fifty percent of patients identified as high-risk using the 12-item *Stay Independent* questionnaire reported falling in the last year, compared to 39% of those identified as high-risk using the *three key questions*.

**Table 2.** Patient Characteristics for Participants Aged 65 and Older by Risk Level Using *Stay Independent* and *Three Key Questions* (2014)

Variable	Low-risk using both approaches (*n* = 124)	Low-risk using *Stay Independent* but yes to any key question (*n* = 111)	High-risk using *Stay Independent* (*n* = 170)	Overall^a^ (*n* = 405)
Weighted percent in each group	63.6%	14.4%	22%	100.0%
Age (mean)	71.8	73.5*	76.5*	73.1
Gender (% female)	61.3%	70.3%	68.8%	64.2%
Race/ethnicity (% white)	95.0%	95.5%	92.2%	94.5%
Comorbid condition prevalence				
Number of comorbidities^b^ (mean)	1.8	2.3*	3.8*	2.3
Arthritis	37.9%	51.4%*	54.1%*	43.4%
Vision problems	36.3%	48.6%	57.6%*	42.7%
Chronic pain	37.1%	48.6%	54.1%*	42.5%
Depression	27.4%	26.1%	38.8%*	29.7%
Diabetes-neuropathy	19.4%	15.3%	33.5%*	21.9%
Muscle weakness-deconditioning	0.8%	2.7%	22.9%*	5.9%
Gait abnormality	1.6%	8.1%*	15.3%*	5.5%
Use of assistive device	0.0%	0.0%	18.8%*	4.1%
Orthostatic hypotension	0.8%	0.9%	8.8%*	2.6%
Percent reporting no comorbidities	13.7%	7.2%	1.2%*	10.0%
*Stay Independent* questionnaire individual item responses				
Mean number positive (of 12 items)	.8	1.9*	5.4*	2.0
1—fell in last year^c^	0.0%	39.4%*	50.3%*	16.5%
2—advised to use cane or walker	0.8%	0.9%	44.4%*	10.3%
3—feels unsteady^c^	0.0%	41.3%*	72.2%*	21.6%
4—holds onto furniture to steady	2.4%	7.3%	45.0%*	12.4%
5—worried about falling^c^	0.0%	28.4%*	58.6%*	16.8%
6—push w/hands to stand from chair	9.7%	11.0%	59.8%*	20.8%
7—trouble stepping onto curb	4.0%	2.8%	40.8%*	11.9%
8—rushes to toilet	16.1%	16.5%	50.9%*	23.8%
9—lost feeling in feet	13.7%	8.3%	36.1%*	17.8%
10—medicine makes me light-headed	7.3%	10.1%	27.2%*	12.0%
11—medicine for sleep or mood	21.8%	20.2%	39.6%*	25.5%
12—feel sad or depressed	6.5%	8.3%	19.5%*	9.6%
% Yes to 1, 3, or 5 (“key questions”)	0.0%	100.0%*	94.7%*	34.9%

^a^Means and percentages for overall category are weighted to account for sampling design (i.e., those in concordant low group were sampled 1:4, and given a weight of 4).

^b^Only the most prevalent comorbidities are listed. See methods for full list of comorbidities.

^c^Three key questions.

*p ≤.05 compared with the concordant low group (reference).

## Discussion and Implications

This study reports the adoption of CDC’s STEADI initiative in an academic primary care clinic and its effect on patient care. Screening rates were moderate, with 64% of eligible patients screened over 6 months, and 22% of screened patients were identified as high-risk for falls. Two-thirds of high-risk patients received additional fall risk assessments and interventions. Many high-risk patients had multiple fall risk factors identified, and most received recommended assessments and interventions.

The implementation was not without challenges. Nearly all (94%) high-risk patients took a medication that increased fall risk, yet only 22% had a medication change. This finding is consistent with other literature that found polypharmacy and high-risk medications to be challenging for PCPs to address (Phelan, Aerts, Dowler, Eckstrom & Casey, 2016). Future research should identify better ways to address medication reduction to reduce fall risk. Additionally, the majority of high-risk patients whose STEADI visit was deferred did not receive further fall-related assessments and interventions during the study period, despite a specific workflow meant to assist staff and providers in scheduling patients for a future fall-focused visit.

The implementation of STEADI allocated patients into high- or low-risk based on the results of the 12-question *Stay Independent* questionnaire. Our analysis showed that using only the *three key questions* identified 95% of these high-risk patients, potentially reducing the time needed to screen patients. However, using the *three keys questions* would have resulted in an additional 111 high-risk patients requiring additional follow-up. We do not have data to determine the potential benefit of targeted follow up with these additional potentially “high-risk” patients. Nor do we know how much time such follow up would take. One benefit of the full *Stay Independent* questionnaire is that responses to individual questions can help the PCP identify specific fall risks. In the absence of a gold standard screening questionnaire that achieves both clinical utility and maximal efficiency, additional research is needed to ascertain the true positive and negative predictive value of these approaches.

### Limitations

Screened patients may not have been representative of the older adult population since providers came from a volunteer sample and participating providers did not screen all eligible patients or evaluate all high-risk patients. This fact could bias the results toward greater uptake of the intervention. Second, it was difficult to identify whether patients who received some fall-risk reduction recommendations (such as participating in community tai chi classes) carried through on these recommendations. Finally, the data collection period was 6 months, so interventions were still underway for many patients, and we were unable to report on health outcomes, such as fall rates. Anecdotally, providers expressed gratitude for having an evidence-based clinical pathway at their fingertips to offer resources and make recommendations to high-risk patients. Importantly, although not formally studied, patients reported satisfaction with STEADI, and for those who adhered to recommended interventions, a belief that the interventions decreased their fall risk.

This study showed that CDC’s STEADI can be adopted in a busy primary care practice. With the STEADI algorithm embedded into the clinic workflow and EHR, PCPs and their clinical teams could consistently implement recommended interventions. Future work should address whether additional strategies could further streamline the process to improve feasibility and how other team members might contribute to the process (e.g., having a pharmacist do the medication review). More sophisticated tracking and follow up could help ensure that high-risk patients with “deferred” visits receive additional interventions and ensure that recommendations for community fall prevention classes and other interventions are followed. Fall prevention remains one of the biggest public health and medical challenges in caring for older adults. Projects such as ours demonstrate how primary care practices can systematically implement an evidence-based algorithm to address fall risk among older adults, and ultimately reduce falls and fall-related injuries.

## Supplementary Material

Supplementary data is available at *Innovation in Aging* online.

igx028_suppl_Supplementary_Figure_1

## Funding

This work was supported by the Health Resources and Services Administration (HRSA) of the US Department of Health and Human Services (HHS) [grant number UB4HP19057] titled “Oregon Geriatric Education Center” (total award amount of $2,138,357, 0% financed with nongovernmental sources). This information or content and conclusions are those of the author and should not be construed as the official position or policy of, nor should any endorsements be inferred by HRSA, HHS or the US Government.

## Conflict of Interest

Elizabeth Eckstrom was funded by HRSA grant #UB4HP19057 and a CDC Intergovernmental Personnel Act Agreement. Elizabeth Eckstrom receives modest royalties for the book “The Gift of Caring: Saving our Parents from the Perils of Modern Healthcare.” Colleen Casey was funded by HRSA grant #UB4HP19057 and a CDC Intergovernmental Personnel Act Agreement. Other authors reported no conflict of interest. The study sponsor had no role in study design; collection, analysis, and interpretation of data; writing the report; and the decision to submit the report for publication. No other financial disclosures were reported by the authors of this paper.
