# Supplementary figures and images for: Implementing STEADI in Academic Primary Care to Address Older Adult Fall Risk
Source: Innov Aging. 2017 Nov 28;1(2):igx028. doi: 10.1093/geroni/igx028 (PMC6016394; doi:10.1093/geroni/igx028)

Supplementary Figure 1. STEADI Algorithm


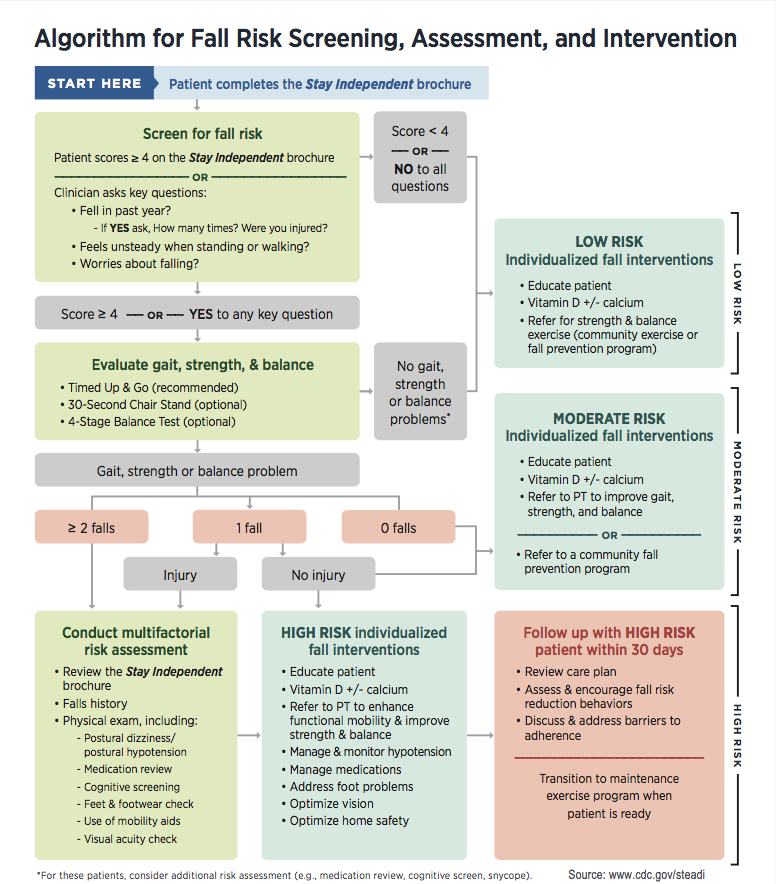

Supplement: igx028_suppl_Supplementary_Figure_1 [file igx028_suppl_supplementary_figure_1.docx]
